# Oxidative Stress and Inflammation Levels in a Population of Eastern European Naïve Versus Treated Psoriasis Patients

**DOI:** 10.7759/cureus.48177

**Published:** 2023-11-02

**Authors:** Elena Codruța Cozma, Mihnea-Alexandru Găman, Olguța Orzan, Kord-Varkaneh Hamed, Vlad Mihai Voiculescu, Amelia-Maria Găman

**Affiliations:** 1 Dermatology, University of Medicine and Pharmacy of Craiova, Craiova, ROU; 2 Hematology, Center of Hematology and Bone Marrow Transplantation, Fundeni Clinical Institute, Bucharest, ROU; 3 Hematology, Carol Davila University of Medicine and Pharmacy, Bucharest, ROU; 4 Dermatology, Elias Emergency University Hospital, Bucharest, ROU; 5 Nutrition, Shahid Beheshti University of Medical Sciences, Tehran, IRN; 6 Dermatology, Carol Davila University of Medicine and Pharmacy, Bucharest, ROU; 7 Medicine, University of Medicine and Pharmacy of Craiova, Craiova, ROU

**Keywords:** redox balance, methotrexate, biotherapies, oxidative stress, antioxidant status, psoriasis

## Abstract

Psoriasis is a chronic inflammatory disease, with a major impact on the patients' quality of life. Oxidative stress (OS) is represented by the imbalance between oxidant and antioxidant mechanisms of the organism, with increased levels being described in the majority of chronic diseases. We present the first prospective study in Romania to evaluate the redox balance changes (using a CR3000 analyzer) in patients with moderate-severe psoriasis based on treatment regimens: treatment-naïve (A), treatment with novel targeted agents (B) and methotrexate (C). The study group included 53 Caucasian patients divided into three groups (A- 27 patients, B - 15 patients, and C - 11 patients) for which OS, antioxidant status, standard blood count, and inflammatory status were evaluated. Our findings demonstrate that patients with psoriasis display high levels of OS, with elevated Free Oxygen Radical Test (FORT) (p-value for group A (pA)<0.0001, p-value for group B (pB)=0.0019 and p-value for group C (pC)=0.0063) and reduced Free Oxygen Radical Defense (FORD) (pB=0.018) values noted in our subjects. Higher erythrocyte sedimentation rate (ESR) values were detected in groups B (pB=0.00012) and C (pC<0.00001). Psoriasis treatments alleviate FORT and FORD levels, but their impact is not sufficient to restore the oxidative balance to normal ranges. Moreover, despite adequate treatment, patients with psoriasis display elevated inflammation levels. Future research should explore in more detail the interplay between OS and inflammation in psoriasis, namely the long-term impact on the redox balance of biotherapies.

## Introduction

Psoriasis is a chronic inflammatory disease characterized by the development of erythematous, indurated, slightly itchy, well-defined plaques covered by thick scales, especially in areas of extension, pressure, or repeated traumatism [1]. Although it was considered a disorder with a strictly cutaneous localization, novel insights into dermatology have delineated the concept of “psoriatic disease.” More categorically, psoriasis is associated with a myriad of comorbidities and/or disease-related complications: psoriatic arthritis, obesity, cardiovascular disease, metabolic syndrome, atherosclerosis, and last but not least gastrointestinal and neuropsychiatric complications [1,2]. Psoriasis remains a frequently diagnosed (78.9-230 cases per 100,000 adult inhabitants) and potentially debilitating skin disorder with notable socioeconomic costs which has attracted the attention of researchers and physicians alike due to its intricate pathophysiology [3,4]. Recent advances in deciphering the complex molecular mechanisms involved in the onset and evolution of psoriasis have been translated from bench to bedside and have allowed for the development of biotherapies, i.e., monoclonal antibodies directed against some key interleukins (IL) involved in the pathogenesis of the disease: IL-12, IL-17, IL-23 and tumor necrosis factor-alpha (TNF-alpha). Biological agents are now widely used in psoriasis management alongside systemic medications (cyclosporine, methotrexate) and have notably changed the evolution of the disease and its impact on the quality of life of affected patients [5].

Oxidative stress (OS) is defined as an imbalance between the production of reactive oxygen (superoxide and hydroxyl radicals, hydrogen peroxide and singlet oxygen) and nitrogen species and the ability of antioxidant systems (superoxide dismutase, catalase, glutathione peroxidase) to scavenge them [6]. OS seems to play a key role in the pathogenesis of chronic diseases such as atherosclerosis, cardiovascular disorders, diabetes, neurodegenerative and autoimmune diseases, as well as in solid and blood cancers. Moreover, several investigations have highlighted its contribution to the onset of several chronic dermatological diseases, e.g., psoriasis, atopic dermatitis, chronic eczema, and others [7]. Furthermore, the association of psoriasis with comorbidities and disease-related complications seems to result in alterations of the redox balance, bringing to the fore the role of OS in the concept of “psoriatic disease.” At the same time, the use of several antipsoriatic drugs has been linked to changes in the redox balance based on the pharmacological agent’s mechanism of action and the type of treatment prescribed, i.e., conventional systemic medicine versus biologics. Researchers have pointed out that some biotherapies increase the antioxidant status and reduce OS, whereas others increase the antioxidant status and maintain or increase OS, whereas classical agents (methotrexate) are strongly pro-oxidant and elevate OS levels [8].

Therefore, the aim of this study was to evaluate OS levels in patients with moderate-severe psoriasis (Psoriasis Assessment Severity Index (PASI) over 10 and Disease Life Quality Index (DLQI) over 10 points) based on sociodemographic, clinical, and laboratory characteristics, presence of comorbidities/disease-related complications and treatment patterns [9].

## Materials and methods

To our knowledge, this is the first prospective observational study in Romania to evaluate the redox balance changes in patients with moderate-severe psoriasis based on treatment regimens: treatment-naive, treatment with methotrexate and treatment with novel targeted agents.

We conducted a hospital-based study over a period of six months (December 2022 to May 2023) in which we included patients with moderate-severe psoriasis (PASI over 10 points and DLQI over 10 points) who attended the Dermatovenereology Department of the Elias University Emergency Hospital, Bucharest, Romania.

Study group and protocol

We included consecutive subjects based on a non-random (convenience) sampling method. The study group included 53 Caucasian patients (Fitzpatrick skin phototypes II-III) diagnosed with moderate-to-severe forms of psoriasis. The recruited subjects were divided into three groups as follows: group A (27 patients) was treatment-naive, group B (15 patients) received treatment with systemic biotherapies, and group C (11 patients) was prescribed systemic treatment with methotrexate. The control group consisted of 30 healthy volunteers with similar characteristics to the study group. The inclusion and exclusion criteria are reported in Table 1. Healthy volunteers who signed the informed consent and who had similar characteristics to the study participants were enrolled in the control group.

**Table 1:** Inclusion and exclusion criteria for study enrollment. *Chronic smoking was not included among the exclusion criteria due to its high prevalence in the investigated population

Inclusion criteria	Exclusion criteria
Histopathological confirmation of psoriasis	Absence of histopathological confirmation of psoriasis
Moderate-to-severe forms of psoriasis classified using PASI and Body Surface Area (BSA) scores	Chronic alcohol consumption*
For group A: patients who did not receive treatment for at least 1 month prior to study enrollment 2	Severe chronic disorders known to be associated with elevated OS levels: cancer, severe heart failure, severe hepatic and renal failure, autoimmune diseases
Patients who had received systemic treatment (methotrexate or biotherapies) for at least three months prior to study enrollment (for groups B and C, respectively)	Psychiatric illnesses that prevented the patients to understand the purpose of the research and who could not sign the informed consent form and accept study participation
Patients agreed to partake in the research and signed the informed consent form	Age <18 years old
Adult patients (age ≥ 18 years)	Pregnancy

oeWe evaluated the following:

- OS by the Free Oxygen Radical Test (FORT) and Free Oxygen Radical Defense (FORD) assays

- chronic inflammation by measuring fibrinogen, C-reactive protein (CRP), and erythrocyte sedimentation rate at 1 hour (ESR 1h)

- standard hematological (complete blood count) and biochemical parameters (fasting plasma glucose, total cholesterol, triglycerides, uric acid and creatinine levels)

- psoriasis severity using the PASI and the impact of the disease on quality of life using the DLQI

All the collected variables (demographics, clinical findings, and laboratory parameters) were entered by the main investigator into a Microsoft Office Excel 2013 Spreadsheet which was further used for data analysis.

Ethical aspects

The present study was carried out in agreement with the national and international research ethics guidelines and was approved by the Ethics Committee of the University of Medicine and Pharmacy of Craiova (approval no. 42/02.04.2021) and of the Elias University Emergency Hospital of Bucharest (approval no. 1092/17.02.2023), Romania. Study enrollment and data collection (demographics, clinical and laboratory data, collection of biological samples, dermoscopic examinations etc.) were carried out only after a preliminary stage in which potentially eligible subjects were informed regarding all aspects and procedures required for the execution of the study, including potential side effects, and after the recruited individuals signed the written informed consent to partake in the research. During the duration of the project, the investigators respected all ethical and medical deontology guidelines both at an institutional level (the code of ethics issue by the University of Medicine and Pharmacy of Craiova) and national level, e.g., The Code of Medical Ethics of 06.06.1997, law no. 319/06.08.2003 regarding the status of research and development personnel, law no. 206/27.05.2004 regarding good conduct in scientific research, technological development and innovation. In this assessment, patients' rights were respected in agreement with the guidelines of the World Health Organization - Law of Patients' Rights no. 46/2003 and the Declaration of Helsinki adopted in 1964 and revised in 1975 and 2002, respectively. Protection of personal patient data was ensured in accordance with the law no. 190/2018 on implementing measures to Regulation (EU) 2016/679 of the European Parliament and of the Council of April 27, 2016 on the protection of natural persons with regard to the processing of personal data and on the free movement of such data and repealing Directive 95/46/EC (General Data Protection Regulation).

Measurement of OS levels

OS assessment in both study and control group, respectively, was carried out using a CR3000 analyzer (Callegari SpA, Italy) in the Laboratory of Oxidative Stress Evaluation, University of Medicine and Pharmacy of Craiova, Craiova, Romania. For FORT evaluation, the principal investigator collected 20 microliters of capillary blood, whereas for FORD assessment 50 microliters were collected. Capillary blood was drawn from the pulp of the index finger by puncturing with a 20-gauge needle without any pressure applied to the finger and after the first drop of capillary blood was discarded. FORT and FORD assessment is based on colorimetric tests. Quantification of these OS parameters in capillary blood is based on the spectral absorbance of the collected sample at different wavelengths (505 nm). The CR3000 analyzer assesses these variables by means of a spectrophotometer.

The FORT assay exploits the property of transition metals (iron) to catalyze the chemical reaction via which transformation of hydroperoxides into radical derivatives occurs, a process denominated as the Fenton reaction. The measurement involves the insertion of the 20-microliter capillary tube containing the collected sample of blood into an acidic buffer which triggers a chemical reaction that results in the transformation of hydroperoxides into free radicals (alkoxyl and peroxyl radicals). The free radicals formed will cause a colorimetric reaction with one of the added reagents (phenylenediamine derivatives), leading to the generation of a chromogenic compound that is detected by the Callegari CR3000 analyzer. The processing of the test specimen and the reading are performed in the first 30 minutes after blood sampling. The process takes on average 15 minutes (nine minutes reagent preparation, six minutes spectrophotometric reading) and all reactions take place at 37°C. The intensity of the color read by the spectrophotometer correlates with the concentration of free radicals in the analyzed sample. The results are delivered in real time, immediately after reading, and are expressed in FORT units (1 FORT unit = 0.26 mg/L hydrogen peroxide (H_2_O_2_)), with normal values of up to 310 FORT units (2.3 mmol/L H_2_O_2_).

The principle of the FORD assay is based on an oxidative reaction that takes place in the presence of ferric chloride (FeCl_3_) in an acidic environment (pH=5.2) which results in the generation of a stable cationic chromogenic product that will be detectable by the spectrophotometer at 505 nm wavelength. The collected blood sample is centrifuged for one minute at 3,000 rotations/minute and then 100 microliters of the resulting supernatant are added to the previously formed chromogen. A secondary reading at 505 nm wavelength is then conducted to assess the decrease in absorbance following the reaction between the antioxidant compounds in the sample (corresponding to the antioxidant systems present in the blood) and the cationic chromophore. Processing and reading are carried out in the first 30 minutes after blood sampling, the process lasting on average 12 minutes (nine minutes preparation of reagents, three minutes spectrophotometric reading), with all reactions taking place at 37°C. Results are expressed in Trolox units (1 Trolox unit = 0.25-0.3 mmol/L). The normal ranges of FORD are 1.07-1.53 mmol/L. Values below 1.07 mmol/L are linked with a decrease in the body's antioxidant mechanisms.

Measurement of inflammatory biological parameters and standard biological parameters

For each patient in the study and control group, respectively, we evaluated several standard laboratory parameters (complete blood count, fasting plasma glucose, lipid profile, creatinine, urea, uric acid), as well as inflammatory markers (ESR1h; fibrinogen; CRP). Measurements were carried out by collecting venous blood after 12 h of overnight fasting before anti-psoriasis treatment initiation. Hematological parameters were measured using the SYSMEX_XN_HEMA analyzer. Biochemical lab values and CRP were evaluated using the ARCHITECT c8000 analyzer. The ACLTOP550H analyzer was used to measure fibrinogen levels. The SEDIPLUS 2000 device was used to determine ESR1h. Laboratory tests were run from venous blood samples and conducted in the medical analysis laboratory of the Elias University Emergency Hospital, Bucharest, Romania. 

In addition, we calculated several parameters highlighted in the literature as being associated with chronic inflammatory responses: neutrophil-to-lymphocyte ratio (NLR), monocyte-to-lymphocyte ratio (MLR), lymphocyte-to-monocyte ratio (LMR), platelet-to-lymphocyte ratio (PLR), mean platelet volume/platelet count ratio (MPV/P), systemic inflammatory index (SII) (neutrophils x platelets/lymphocytes). 

NLR is considered a novel hematological marker used to assess the interrelationship between the innate and adaptive immune response in different pathological conditions, including chronic inflammatory disorders, cardiovascular disease, chronic lung diseases, infectious diseases, solid or hematological cancers, stress etc. It is considered an independent predictor of mortality from any cause, but clear normal values have not yet been established, the limits of the normal range varying from one study to another and being influenced by age and sex. Some studies mention the normal range between 0.7 and 3 [10].

MLR and LMR are considered novel hematological markers as well. They evaluate the interrelation between lymphocytes and monocytes which play a role in cancer progression and suppress the activity of lymphocytes. Increased values have been noted in solid tumors (prostate cancer, breast cancer), myocarditis, pneumonia, tuberculosis, lymphoma, rheumatoid arthritis, etc. [11,12]. 

PLR is another easy to evaluate parameter which is considered a novel marker of inflammation. Elevated PLR values have been reported in inflammatory conditions, as well as metabolic and neoplastic processes in relationship with the role played by platelets in inflammation. PLR seems to correlate with NLR and other inflammation markers [13].

SII is a complex parameter obtained by calculating the ratio between (neutrophils x platelets) and lymphocytes. SII evaluates the interrelation between the immune and the inflammatory response, respectively. Initially emerging as a marker of poor prognosis in cancers, SII has also been used in the assessment of inflammatory and autoimmune diseases [14].

Assessment of psoriasis severity and impact on quality of life

Psoriasis severity and the impact of the disease on the quality of life were assessed by the PASI and DLQI scores, respectively. The PASI score is used to evaluate psoriasis severity and takes into account the presence of erythema, induration, desquamation and the affected surface in the four regions of the body (head, trunk, upper limbs, lower limbs). PASI score values range from 0 to 72 points. Values below 5 are considered to be associated with mild forms, values between 5 and 10 indicate moderate forms, and scores above 10 are associated with severe forms of psoriasis. The PASI score was assessed at the first presentation by the same investigator.

The DLQI score is used to evaluate the impact of the disease on the patient's quality of life. The subject answers 10 questions whose responses highlight if psoriasis symptoms and specific treatment have impacted the patient's daily activities (work, recreational activities, relationships) in the last seven days. The score can range from 0 to 30 points, with DLQI>10 points being associated with a meaningful impact of the disease on the subject’s quality of life regardless of the PASI value.

Statistical analysis

Independent sample t tests and analysis of variance were run to assess differences between selected variables, respectively. Pearson/Spearman correlations were performed to evaluate the relation of FORT, FORD, PASI and DLQI with selected variables. All statistical analyses were performed using SPSS Statistic Software (version 25.0, IBM Corp., Armonk, NY). A p-value <0.05 was considered as the significance level.

## Results

The study group included 53 Caucasian patients diagnosed with psoriasis and was divided into three subgroups (A, B, C) based on the treatment prescribed: A - treatment-naive (no treatment prescribed), B - patients who were prescribed biotherapies, and C - patients who were prescribed systemic treatment with methotrexate. The general characteristics of each subgroup are presented in Table 2. Intragroup and intergroup comparisons are presented in Table 3. FORT and FORD variations based on associated comorbidities are depicted in Table 4.

**Table 2:** Selected characteristics of cases and controls participating in the study. Data are presented as Mean ± SD (FORT, FORD, Glucose, Cholesterol, Tryglicerides, Hemoglobin, Uric acid, ESR1h, Fibrinogen, CRP, Lymphocytes, Monocytes, Neutrophils, WBC, NLR, MLR, PLR, SII, MPV/P, MPV, PASI, DLQI) or n (%) (Age, Sex). FORT – free oxygen radical test; FORD – free oxygen radical defense; ESR1h – erythrocytes sedimentation rate at 1 hour; CRP – C reactive protein; WBC – white blood cells; NLR – neutrophils/lymphocytes ratio; MLR – monocytes/lymphocytes ratio; PLR – platelet/lymphocytes ratio; SII – systemic inflammatory index; MPV/P – mean platelet volume/platelet count ratio; MPV – mean platelet volume; PASI – psoriasis assessment severity index; DLQI – daily life quality index.

Characteristic	Group A (n=27)	Group B (n=15)	Group C (n=11)		Controls (n = 30)	Normal range
Age (years)	45.47±15.82	47±14.99	54.27 ±17.45	52.73 ± 17.42		-
Sex (female)	59.25% (16)	46.66% (7)	45.45% (5)	63.3% (19)		-
FORT (FORT units)	342.59±133.72	268.87±88.33	279.09± 80.84	273 ± 21.09		<310
FORD (mmol/L Trolox)	0.44±0.25	0.63±0.35	0.7±0.45	1.27±0.134		1.07-1.53
Glucose (mg/dL)	109.85±74.02	100.33±28.41	89.73±8.36	92.63± 7.59		70-115
Cholesterol (mg/dL)	201.81±48.45	210.33±51.52	201±35.58	137.70±20.45		140-200
Triglycerides (mg/dL)	107.93±56.53	115.2±61.98	104.27±36.68	95.77±20.22		35-150
Hemoglobin (g/dL)	14.35±1.33	14.57±1.37	13.96±1.12	13.27±1.01		13.1-16.9
Uric acid (mg/dL)	10.37±22.94	5.50±1.71	5.58±1.55	3.80±0.92		3.5-7.2
ESR1h (mm/1h)	18.3±12.05	20.27±16.79	15.64±8.33	5.73±1.78		0-15
Fibrinogen (mg/dL)	371.93±57.84	357.13±75.34	331.27±49.47	267.97±37.26		238-498
CRP (mg/dL)	5.54±4.41	4.81±5.98	3.98±4.24	3.24±0.59		<10
Lymphocytes (x10^3^/μL)	28.3±6.44	26.62±7.27	25.74±3	1.92±0.374		0.6-3.4
Monocytes (x10^3^/μL)	0.58±0.21	0.59±0.16	0.51±0.13	0.46±0.11		0-0.9
Neutrophils (x10^3^/μL)	5.12±1.62	4.99±1.38	4.4±0.9	4.54±0.84		1.5-6.9
WBC (x10^3^/μL)	8.1±1.9	7.79±1.45	6.77±1.12	6.93±1.24		4.6-10.2
NLR	2.42±1.12	2.64±1.12	2.55±0.61	2.35±0.22		-
MLR	0.27±0.11	0.31±0.14	0.3±0.07	0.234±0.058		-
PLR	134.58±36.72	165.15±42.95	147.73±33.48	144.10±32.41		-
SII	698.68±373.9	843.87±387.31	649.38±208.99	651.37±179.36		-
MPV/P	0.04±0.01	0.04±0.01	0.04±0.01	0.034±0.010		-
MPV (/fL)	10.68±1.04	10.53±1.04	10.96±0.79	8.86±1.78		6.7-11.5
PASI (points)	13.98±12.32	7.9±10.06	9.84±7.23	0		0
DLQI (points)	10.85±7.13	4.87±4.94	8.64±7.54	0		0

**Table 3:** Calculated p-values (using independent t-test) for the evaluated parameters for each studied group in comparison with the control group, and between the groups. ^a^Independent samples t-test was used for continuous variables and Chi-square test was used for categorical variables. FORT – free oxygen radical test; FORD – free oxygen radical defence; ESR1h – erythrocytes sedimentation rate at 1 hour; CRP – C reactive protein; WBC – white blood cells; NLR – neutrophils/lymphocytes ratio; MLR – monocytes/lymphocytes ratio; PLR – platelet/lymphocytes ratio; SII – systemic inflammatory index; MPV/P – mean platelet volume/platelet count ratio; MPV – mean platelet volume; PASI – psoriasis assessment severity index; DLQI – daily life quality index.

Evaluated parameter	Compared groups (p^a)^ values)					
	A-control	B-control	C-control	A-B	A-C	B-C
Age (years)	0.059	0.141	0.401	0.132	0.075	0.132
FORT (FORT units)	0.00001	0.0019	0.0063	0.033	0.040	0.382
FORD (mmol/L Trolox)	0.090	0.018	0.180	0.170	0.440	0.210
Glucose (mg/dL)	0.109	0.080	0.140	0.310	0.189	0.120
Cholesterol (mg/dL)	0.033	0.00001	0.00001	0.000151	0.00171	0.03
Triglycerides (mg/dL)	0.137	0.061	0.174	0.350	0.422	0.303
Hemoglobin (g/dL)	0.167	0.105	0.383	0.323	0.340	0.234
Uric acid (mg/dL)	0.0002	0.0016	0.0008	0.41	0.46	0.35
ESR1h (mm/1h)	0.070	0.00012	0.00001	0.0031	0.527	0.204
Fibrinogen (mg/dL)	0.011	0.00001	0.00004	0.0006	0.0079	0.166
CRP (mg/dL)	0.0001	0.326	0.016	0.000091	0.009	0.081
Lymphocytes (x10^3^/μL)	0.0001	0.0018	0.01	0.188	0.266	0.099
Monocytes (x10^3^/μL)	0.00001	0.00001	0.00023	0.345	0.349	0.339
Neutrophils (x10^3^/μL)	0.00001	0.00025	0.0021	0.208	0.2107	0.094
WBC (x10^3^/μL)	0.00001	0.0004	0.0022	0.446	0.48	0.441
NLR	0.0001	0.0001	0.0001	0.299	0.210	0.360
MLR	0.171	0.152	0.0577	0.430	0.239	0.310
PLR	0.240	0.327	0.239	0.440	0.387	0.341
SII	0.345	0.108	0.108	0.201	0.191	0.451
MPV/P	0.148	0.414	0.201	0.262	0.476	0.291
MPV (/fl)	0.48	0.499	0.263	0.464	0.311	0.295
PASI (points)	-	-	-	0.000126	0.0005	0.399
DLQI (points)	-	-	-	0.392	0.098	0.068

**Table 4:** Comparing FORT and FORD values according to comorbidities ^a^Independent samples t-test was used for continuous variables and Chi-square test was used for categorical variables FORT – free oxygen radical test; FORD – free oxygen radical defense; CHD – chronic heart disease.

Characteristic		FORT (Mean ± SD)	P-value^a^	FORD (Mean ± SD)	P-value
Psoriasis arthritis	yes	306.07±119.88	0.564	0.581±0.354	0.006
	no	324.86±95.80		0.328±0.130	
Hypertension	yes	301.65±123.63	0.365	0.558±0.314	0.291
	no	321.16±104.04		0.528±0.398	
Diabetes	yes	299.98±114.51	0.750	0.562±0.350	0.115
	no	413.50±94.58		0.367±0.1912	
Obesity	yes	295.32±121.90	0.539	0.584±0.327	0.870
	no	327.18±108.03		0.495±0.365	
Dyslipidemia	yes	311.97±121.51	0.737	0.559±0.353	0.703
	no	299.87±105.58		0.518±0.325	
CHD	yes	304.15±120.18	0.226	0.535±0.317	0.035
	no	350.80±61.72		0.670±0.573	
Medication that decreases the oxidative stress	yes	291.97±109.94	0.362	0.549±0.325	0.458
	no	350.53±125.31		0.544±0.397	
Other autoimmune diseases that increase oxidative stress	yes	286.95±116.73	0.011	0.565±0.329	0.489
	no	382.33±82.290		0.486±0.395	

Significantly higher values of FORT were observed for each of the three subgroups compared to the control group: group A vs control - 342.59 ± 133.72 FORT units vs. 273 ± 21.09 FORT units, p<0.0001; group B vs control 268.87 ± 88.33 FORT units vs 273 ± 21.09 FORT units, p=0.0019; group C vs control - 279.09 ± 80.84 FORT units vs 273 ± 21.09 FORT units, p=0.0063. Moreover, treatment with biotherapies and methotrexate, respectively, significantly decreased FORT levels (P=0.033 for biotherapies and p=0.04 for methotrexate, respectively). Significantly lower FORD levels were observed in patients who were managed using biotherapies compared to the control group (0.63±0.35 mmol/L vs 1.27±0.134 mmol/L, p=0.018).

Significantly higher total cholesterol concentrations were observed in all analyzed subgroups (A, B, and C) compared to controls (A: 201.81±48.45 mg/dL vs 137.70±20.45 mg/dL, p=0.033; B: 210.33±51.52 mg/dL vs 137.70±20.45 mg/dL, p<0.00001; C: 201±35.58 mg/dL vs 137.70±20.45 mg/dL, p<0.0001). Total cholesterol values decreased significantly after treatment with biotherapies p=0.00015) and methotrexate (p=0.0017), respectively. Serum uric acid levels were significantly elevated in patients with psoriasis, regardless of treatment status (A: 10.37±22.94 mg/dL vs 3.80 ±0.92 mg/dL, p=0.0002; B: 5.50±1.71mg/dL vs 3.80 ±0.92 mg/dL, p=0.0016; C: 5.58±1.55 mg/dL vs 3.80 ±0.92 mg/dL, p=0.0008).

In terms of inflammation markers, higher ESR1h values were detected in group B (20.27±16.79 mm/h vs 5.73±1.78 mm/h, p=0.00012) and C (15.64±8.33 mm/h vs 5.73±1.78 mm/h, p<0.00001), respectively, compared to the control group. Treatment with biotherapies significantly increased ESR1h levels in psoriasis patients vs treatment-naive subjects (A: (20.27±16.79 mm/h vs 18.3±12.05 mm/h, p=0.0031). Fibrinogen concentrations were notably elevated in subgroups A and B and C versus controls (A: 371.93±57.84 vs 267.97±37.26 mg/dL, p=0.011; B: 357.13±75.34 mg/dL vs 267.97±37.26 mg/dL, p<0.00001; C: 331.27±49.47 mg/dL vs 267.97± 37.26 mg/dL, p=0.00004). Patients who received treatment with biotherapies (p=0.0006) and methotrexate (p=0.0079) exhibited notable decreases in fibrinogen levels versus treatment-naive subjects. CRP concentrations were significantly elevated (5.54±4.41 mg/L vs 3.24±0.59 mg/L, p=0.0001) in patients with psoriasis as compared to the control group. Individuals diagnosed with psoriasis displayed elevated lymphocyte, neutrophil and monocyte counts compared to healthy controls, irrespective of the treatment-naive status or type of treatment prescribed (p<0.0001 for group A vs controls for all variables). NLR values were notably higher in patients with psoriasis compared to the control group (A: 2.42±1.12 vs 2.35±0.22, p=0.0001; B: 2.64±1.12 vs 2.35±0.22, p=0.0001; C: 2.55±0.61 vs 2.35 ±0.22, p=0.0001). Differences remained significant even after the subjects received treatment with biotherapies or methotrexate.

We observed statistically significant positive associations between FORT and glucose concentrations (Pearson correlation coefficient (r)=+0.310, p=0.024), ESR1h (r=+0.345, p=0.011), and MPV (r=+0.276, p=0.046), whereas FORD was positively correlated with MPV/P (r=+0.284, p=0.039) and total bilirubin (r=+0.363, p=0.007) levels in patients with psoriasis. A negative association was noted between FORT and PLR (r=-0.328, p=0.017) in subjects with psoriasis. In the multivariate analysis, glucose (F=16.54, p<0.001), uric acid (F=242.987, p<0.001), creatinine (F=10.983, p<0.001) and aspartate aminotransferase (F=13.090, p<0.001) levels emerged as predictors of FORT values.

## Discussion

Our study focused on the evaluation of OS levels, namely the total antioxidant capacity assessed by the FORD assay and the pro-oxidant status assessed by the FORT assay, in patients with moderate-severe psoriasis versus healthy controls. In addition, we investigated the impact of antipsoriatic treatment on OS, inflammation, and other laboratory parameters based on data derived from three patient subgroups: treatment-naive (group A), systemic treatment with targeted therapies (group B) or with methotrexate (group C). Moreover, we evaluated the impact of chronic comorbidities on the aforementioned variables. To our knowledge, this is the first study from Romania to assess OS using the FORT and FORD assays and to evaluate the impact of treatment-naive status and antipsoriatic agents on OS.

Treatment impacts OS levels in psoriasis

Evaluation of OS levels by FORT measurement highlighted statistically significant differences between subjects diagnosed with psoriasis and healthy controls, as well as between treatment-naive and psoriasis patients on biotherapies or methotrexate (p<0.00001 for group A vs controls, p=0.0019 for group B vs controls, p=0.0063 for group C vs controls). Our findings suggest an alteration of the pro-oxidative balance in individuals with psoriasis, regardless of the treatment status. At the same time, the comparison of groups A and B and A and C, respectively, revealed statistically significant differences between patient subgroups who were prescribed therapy (B and C) and treatment-naive subjects (group A). Thus, our results point out that antipsoriatic agents improve oxidative balance; however, they do not impact OS sufficiently to restore it to levels similar to those detected in the healthy population. Although there are numerous investigations in the literature that have evaluated OS involvement in psoriasis, there is still limited evidence on the impact of anti-psoriasis therapy on OS markers.

Our findings are consistent with the results of several previously published assessments. Thus, Skutnik-Radziszewska et al., Kizilyel et al., and Esmaili et al., respectively, have conducted case-control studies in which they evaluated numerous OS markers using venous blood samples collected from psoriasis subjects and compared the results to data obtained from control groups consisting of healthy individuals. Among the evaluated parameters, the total oxidative status (TOS) and reactive oxygen species concentrations (ROS) were the most important markers of OS measured. Each of the aforementioned researchers observed significantly higher values of TOS and ROS in patients with psoriasis versus the healthy population (p<0.001, p<0.001, and p=0.04, respectively) [15-17]. In terms of the impact of systemic therapies on oxidative balance, previous investigations have led to conflicting results. In our study, we observed a decrease in OS levels in patients treated with methotrexate versus treatment-naive individuals (p=0.04). However, psoriasis subjects on methotrexate treatment continued to maintain elevated OS values when compared to healthy controls (p=0.0063). These results might be explained by the pro-oxidant and pro-apoptotic role of methotrexate. In an assessment that included 26 patients with psoriasis, Kilic et al. evaluated the effect of 8 weeks of methotrexate administration on TOS concentrations. However, they did not register notable differences in TOS levels following methotrexate prescription. A possible explanation for these findings is that TOS values were measured too soon after treatment initiation [18]. In our case, we measured OS markers at least three months after starting the therapy and we demonstrated that antipsoriatic agents can decrease OS levels. Regarding subgroup B who was prescribed novel, targeted molecules, our findings are similar to those obtained in subgroup C who received systemic treatment with methotrexate, i.e., statistically significant differences versus subgroup A of treatment-naive subjects and versus healthy volunteers. These data support once again the fact that, despite improvement of disease symptoms and oxidative balance, subjects with psoriasis exhibit elevated OS levels in comparison with the healthy population. 

However, there is limited evidence in the literature regarding the impact of antipsoriatic agents on OS levels. Barygina et al. evaluated the impact of infliximab administration in 47 individuals with psoriasis and reported notable reductions in OS markers (p<0.05). They measured TOS, protein carbonyl groups, and thiobarbituric acid reacting substances concentrations at 6 months after therapy initiation [19]. Campanati et al. evaluated the benefits of a 12-week prescription of etanercept and adalimumab on 12 subjects with psoriasis and observed a decrease in reactive nitrogen species levels in the skin biopsies of these individuals [20].

Another molecule with an important role in the oxidative balance is uric acid which is known to play a key role in elevating ROS concentrations. In our investigation, we highlighted an increase in uric acid values in all the analyzed subgroups (A, B, and C, respectively) versus controls (p=0.0002, p=0.0016, and p=0.0008, respectively). However, no significant improvement in uric acid values was noted after the administration of systemic agents. Similarly, Oszukawska et al. analyzed laboratory data from 66 patients with psoriasis and confirmed that uric acid levels are higher in subjects with this skin condition versus healthy controls (p<0.001) [21].

Impact of treatment on the antioxidant capacity in psoriasis patients

Although patients with psoriasis displayed lower FORD values versus controls (0.547±0.343 mmol/L vs. 1.27±0.134 mmol/L, p<0.001), we observed no significant intergroup differences in subjects with psoriasis who were prescribed therapy. Our findings suggest that, despite the fact that treatment influences disease severity by notably improving PASI scores, it does not improve the capacity of antioxidant systems. This result can be explained by an initial improvement of the oxidative balance via an OS decrease. In this process, antioxidant systems are consumed, and reaching antioxidant levels similar to those detected in the healthy population requires time. Moreover, our data is in accordance with the data from the literature. Thus, Surucu et al. evaluated the total antioxidant status (TAS) in 40 patients with psoriasis versus 47 controls, detecting lower TAS levels in subjects with psoriasis (p=0.01) [22]. Similar findings were reported by Kaur et al. who demonstrated that psoriasis subjects exhibited decreased TAS concentrations (p<0.001) [23]. Furthermore, Houshang et al. evaluated antioxidant systems concentrations (catalase, superoxide dismutase, paraoxonase-1) in 100 patients with psoriasis and highlighted that subjects with this dermatological condition exhibit lower superoxide dismutase, catalase, and paraoxonase-1 concentrations (p<0.05) versus controls [24]. However, the impact of antipsoriatic drugs remains controversial. Elango et al. observed reductions in TAS, catalase, and superoxide dismutase levels in 56 individuals suffering from psoriasis who were administered methotrexate 7.5 mg/week for 12 weeks. This phenomenon might be explained by an increased activity of antioxidant systems and their subsequent consumption as a result of the sustained pro-oxidative effect of methotrexate [25]. Kilic et al. did not observe any statistically significant difference in terms of TAS levels in 26 patients diagnosed with psoriasis who were treated with methotrexate for eight weeks [18]. Regarding biotherapies, Barygina et al. demonstrated an improvement in antioxidant systems, i.e., an increase in TAS concentrations (p<0.05), following 6 months of infliximab administration [19]. Interestingly, Campanati et al. noted a conflicting effect of adalimumab on antioxidant concentrations. After a treatment course of 12 weeks of adalimumab, superoxide dismutase values increased (p<0.05) and catalase values decreased (p<0.05) in the lesional and perilesional skin [20].

Inflammation in psoriasis and the impact of antipsoriatic agents

We also evaluated inflammation markers (CRP, ESR1h, and fibrinogen levels) and detected significantly higher values of these parameters in psoriasis subjects versus controls, in the naive, but also the treated groups. Thus, we discovered that fibrinogen concentrations were significantly elevated in each psoriasis subgroup (A, B, and C) compared to the control group (p=0.011, p<0.00001 and p=0.00004, respectively). Treatment-naive psoriasis subjects had higher fibrinogen values versus patients who were prescribed methotrexate (p=0.0079) and biotherapies (p=0.0006). The difference in ESR1h levels was only statistically significant between patients on systemic therapy versus controls (p=0.00012 for group B vs controls, and p<0.00001 for group C vs controls, respectively). Thus, we may infer that both methotrexate and targeted therapies can play a role in increasing ESR1h values. The results do not completely align with those found in the literature, a possible explanation being the late access to biotherapies of patients with psoriasis in Romania (after a long period of inefficiency of conventional systemic therapy), which determines the administration of biotherapies when the inflammatory process has already been ongoing for a long period of time. Moreover, the provenience of the patients from an urban environment with high pollution indices, associated with the additional presence of psoriasis, may represent an explanation for the increased values of ESR1h [26]. Also, although systemic therapies cause a decrease in inflammatory markers (a decrease compared to the group of untreated patients), their values do not become comparable to those of naïve patients, emphasizing the persistence of background inflammation, despite the treatment. CRP values were also significantly higher in patients with treatment-naive psoriasis (p=0.0001) and psoriasis subjects treated with methotrexate (p=0.016) compared to the control group. CRP levels were lower in individuals with psoriasis who received treatment, however, CRP concentrations remained elevated in psoriasis patients receiving specific therapy versus controls. Thus, patients undergoing systemic treatment with biotherapies and with methotrexate, respectively, exhibited significantly lower CRP values (p=0.000091 and p=0.009, respectively) than patients with psoriasis without treatment. Several studies in the literature support our findings. Kirmit et al. pointed out an increase in CRP levels in patients with psoriasis compared to healthy controls (p=0.04) [27]. Similar changes were observed by Zhou et al. who measured inflammation markers in 214 patients with psoriasis (p<0.001) [28]. Moreover, Balta et al. (p<0.001) reported elevated concentrations of inflammation markers in this dermatological condition [29]. Grechin et al. evaluated the variations of inflammation markers before and after systemic treatment with methotrexate or biotherapies in patients with moderate-severe psoriasis and observed a statistically significant decrease in ESR1h (p<0.001) after administration of methotrexate and notable reductions (p<0.001) in all parameters after treatment with biotherapies [30].

Regarding NLR, MLR, PLR, and SII values, we only detected statistically significant higher NLR values in patients with psoriasis. All patients with psoriasis, irrespective if they were treatment-naive or were already prescribed specific therapy, displayed elevated NLR values (p=0.0001) compared to the healthy control group. NLR has not been intensively investigated in psoriasis, however, elevated values for this parameter have been reported in various chronic inflammatory diseases and its role in immunoinflammation is well-known [10]. Future research is needed to clarify whether elevated NLR values and changes in MLR, PLR, and/or SII values can emerge into a characteristic profile of psoriasis as these parameters are easy to calculate, however, assessment of a larger group of subjects is needed to reach a conclusion.

Changes in PASI and DLQI

Our data are in agreement with the available literature. Previous investigations have highlighted significant decreases in PASI in individuals with psoriasis who were prescribed systemic treatment with methotrexate or biotherapies [31]. However, DLQI was not notably reduced, suggesting that the disorder impacts one’s quality of life even if significant improvement of skin lesions occurs.

Oxidative balance and comorbidities in psoriasis

Psoriasis is associated with a myriad of comorbidities that can potentially alter the redox balance. Consequently, it becomes difficult to evaluate OS levels in subjects suffering from this disorder. However, our study highlighted that psoriatic arthritis, CHD, and autoimmune diseases are the only comorbidities associated with changes in the redox balance in psoriasis. Surprisingly, FORD values were higher in patients with psoriatic arthritis versus those without, and FORT levels were lower in patients with autoimmune disorders versus those without. CHD was associated with lower FORD levels as expected. However, the presence of obesity, cardiovascular disease, dyslipidemia, diabetes mellitus, and hypertension were not linked to significant differences in FORT and FORD levels in psoriasis based on our findings which might suggest that the alteration of the redox balance occurs independently of the presence of cardiometabolic comorbidities [32]. There are no investigations in the literature regarding the impact of associated comorbidities on OS levels in patients suffering from psoriasis, however, it is well-known that ROS concentrations are increased, and the antioxidant capacity is reduced in individuals who suffer from obesity, type 2 diabetes mellitus, hypertension, or cardiovascular disease.

Strengths and limitations of the study

To our knowledge, the current study is the first assessment conducted in Romania to evaluate FORT, FORD, and inflammation markers, as well as their association with several biological parameters (complete blood count, fasting plasma glucose, liver and renal function tests, lipid profile) in three subgroups of patients with moderately severe psoriasis: treatment-naive and patients on systemic treatment with biotherapies and systemic treatment with methotrexate. Moreover, there are no assessments, even at an international level, to evaluate OS and inflammation levels and the impact of targeted molecules (anti-interleukin 17 and anti-interleukin 23 agents) in psoriasis. However, the selection of subjects for subgroups was extremely demanding, particularly for subgroup A for which we recruited individuals who had not received any medicine in the last three months before the evaluation conducted in our study. Moreover, the interplay of the oxidative balance with numerous physiological and pathophysiological processes (nutrition, dietary patterns and use of dietary supplements, smoking, stress, aging) and comorbidities (obesity, cardiovascular disease, diabetes, hypertension, dyslipidemia, autoimmune diseases, etc.), as well as the frequent association of psoriasis with other conditions, made patient selection restrictive. Consequently, the study sample was rather small. Moreover, smoking and pro-oxidant diets have a high prevalence in the Romanian population, and it was thus impossible to exclude patients who adhered to these practices as these exclusion criteria would have markedly decreased the number of recruited individuals and would have eventually led to the impossibility of implementing the research. Another setback is that FORD and FORT assays need to be conducted as soon as possible after capillary blood collection, as ROS gets degraded quickly.

## Conclusions

Psoriasis is a chronic inflammatory disease in whose pathogenesis of OS plays an important role. Our findings demonstrate that patients with this skin disorder display high levels of OS, with elevated FORT and reduced FORD values noted in subjects diagnosed with psoriasis. Although specific treatment with methotrexate or biotherapies alleviates FORT and FORD levels, their impact is not sufficient to restore the oxidative balance to normal ranges. Moreover, psoriasis is associated with increased inflammation levels, as revealed by the changes in the complete blood count, NLR, fibrinogen, CRP, and ESR1h discovered in our assessment. Concurrently, since treatment does not restore the oxidative balance in psoriasis, we consider complementary therapeutic approaches are needed to target this hallmark of psoriasis pathogenesis. Future research is thus required to investigate the role of OS and inflammation in psoriasis pathogenesis and the impact of classical and/or novel therapeutics on these pathophysiological processes, as well as to implement complementary strategies that specifically target OS and inflammation in psoriasis.
